# Flow cytometric discrimination of seven lineage markers by using two fluorochromes

**DOI:** 10.1371/journal.pone.0188916

**Published:** 2017-11-30

**Authors:** Francesco Boin, Maria Letizia Giardino Torchia, Ivan Borrello, Kimberly A. Noonan, Matthew Neil, Mark J. Soloski, Raffaello Cimbro

**Affiliations:** 1 Division of Rheumatology, Johns Hopkins University School of Medicine, Baltimore, Maryland, United States of America; 2 Laboratory of Immune Cell Biology, National Cancer Institute, National Institutes of Health, Bethesda, Maryland, United States of America; 3 Department of Oncology, Johns Hopkins University School of Medicine, Baltimore, Maryland, United States of America; Karolinska Institutet, SWEDEN

## Abstract

Flow cytometry is the primary immunological technique used to analyze multiple parameters on complex cell populations. We present a staining method that identifies major human mononuclear lymphoid and myeloid populations (CD4^+^ and CD8^+^ T cells, γδ T cells, B cells, NK cells and monocytes), using only two fluorochromes and a minimal number of cells. Our approach increases the number of markers recordable on most flow cytometers allowing for a deeper and more comprehensive immunophenotyping.

## Introduction

Flow cytometry is a single-cell resolution technique to simultaneously analyze the expression of multiple parameters at a rate of thousands of events per second[1]. Fluorescence-independent parameters (forward and side scatters) measure the relative cell size and complexity. Fluorescence-dependent parameters, which account for most of the information in flow cytometry data, detect the expression of biological markers by using probes labeled with fluorochromes. Cutting edge immunology requires an increasing number of parameters to identify cell populations (lineage markers) and investigate the expression of biological markers[1]. However, the number of fluorochromes recordable by instruments and inherent optical limitations, such as fluorochrome spectral overlap, restrict the number of parameters/fluorochromes that can be simultaneously detected. As consequence, in several instances flow cytometry analyses are either restricted to a specific cell population, or necessitate separate staining panels to study concurrently different cell lineages. The former approach results in a profound loss of information, while the latter is error prone and difficult to apply when patient samples are limited.

Here, we report a two-fluorochrome immune-cell staining protocol to identify CD4^+^ T cells, CD8^+^ T cells, γδ T cells, B cells, NK cells and classical monocytes that are among the most studied and characterized cellular subsets of the human immune system. Seven distinct lineage markers and fluorochromes (CD3, CD4, CD8, CD14, CD19, CD56 and TCR γδ) are usually required to identify these populations by flow cytometry (www.hcdm.org)[2]. Our approach aggregates these markers in only two fluorochromes by taking advantage of their specific and relatively constant expression pattern on each cell population.

## Materials and methods

### Cell preparation

All studies of human materials were approved by the Johns Hopkins Institutional Review Board under the Health Insurance Portability and Accountability Act. Patient and control samples were de-identified. Peripheral blood mononuclear cells (PBMC) from healthy adult blood donors and patients were isolated from whole blood by LSM gradient centrifugation (LSM; MP Biomedicals). When indicated 10% DMSO cryo-preserved PBMC were first thawed at 37°C, stained and then analyzed by flow cytometry. PBMC and blood from healthy controls were obtained by informed consent.

### Cell staining

For each staining condition 0.5-1x10^6^ PBMC were stained in 96-well V-bottom plate. All the staining procedures were performed at room temperature. Dead cells were labeled for 10 minutes in 100 ul of PBS containing live/dead fixable blue dead cell stain (Molecular Probes). For surface marker staining, cells were labeled for 25 minutes in 30 ul of PBS 0.1% sodium azide containing the relevant antibodies. Description of the specificity, fluorochromes and concentrations of the antibodies that were used can be found in the dedicated section below. We tested different staining buffers (PBS, PBS 0.5% BSA, PBS 0.2% sodium azide or PBS 0.5% BSA 0.1% sodium azide) with comparable results. Samples were also fixed with 2% PFA without loss of signal. When multiple brilliant violet conjugated antibodies were present we used the provided buffer following the manufacturer’s instruction (BD Biosciences). For whole blood staining 200 μl of blood were directly incubated with the staining antibodies for 25 minutes. This was followed by a red cell lysis step with ACK lysis buffer (Life Technologies) following the manufacturer’s instructions. Data were acquired with a FACSAria (Becton Dickinson) equipped with four lasers (355nm-20mW, 405nm-50mW, 488 nm-100mW and 635nm-35mW), and analyzed with FCS Express (De Novo Software). The FCS data, detailed description of the gating strategy and description of antibodies and fluorochromes can be found at https://flowrepository.org/id/FR-FCM-ZYDD.

### Multiple myeloma patients

Study participants were at least 18 years old with a diagnosis of active, symptomatic myeloma requiring treatment (Durie Salmon Stage II or III). Patients were eligible for the study if they had never previously received an autologous stem cell transplant (SCT) and had measurable disease at the time of the marrow infiltrating lymphocytes (MILs) harvest as determined by the presence of a monoclonal spike in their serum and/or urine coupled with a detectable presence of clonotypic plasma cells in the bone marrow. Twenty-two patients gave written informed consent in accordance with the Declaration of Helsinki. Patients then underwent a standard stem cell mobilization with cyclophosphamide and G-CSF. A melphalan-200 preparative regimen (Mel 200) was given in preparation for the stem cell transplant (SCT). Day 0 correspond to the day of the SCT. At day 0, 14, 28, 60, 180, 360 purified PBMC samples were viable cryo-preserved and stored in liquid nitrogen. Frozen cells from all the time points were thawed, stained and analyzed by flow cytometry on the same day. The study was approved by the Institutional Review Board of Johns Hopkins University and registered with Clinicaltrials.gov NCT00566098[3].

## Results

**Fig 1:**
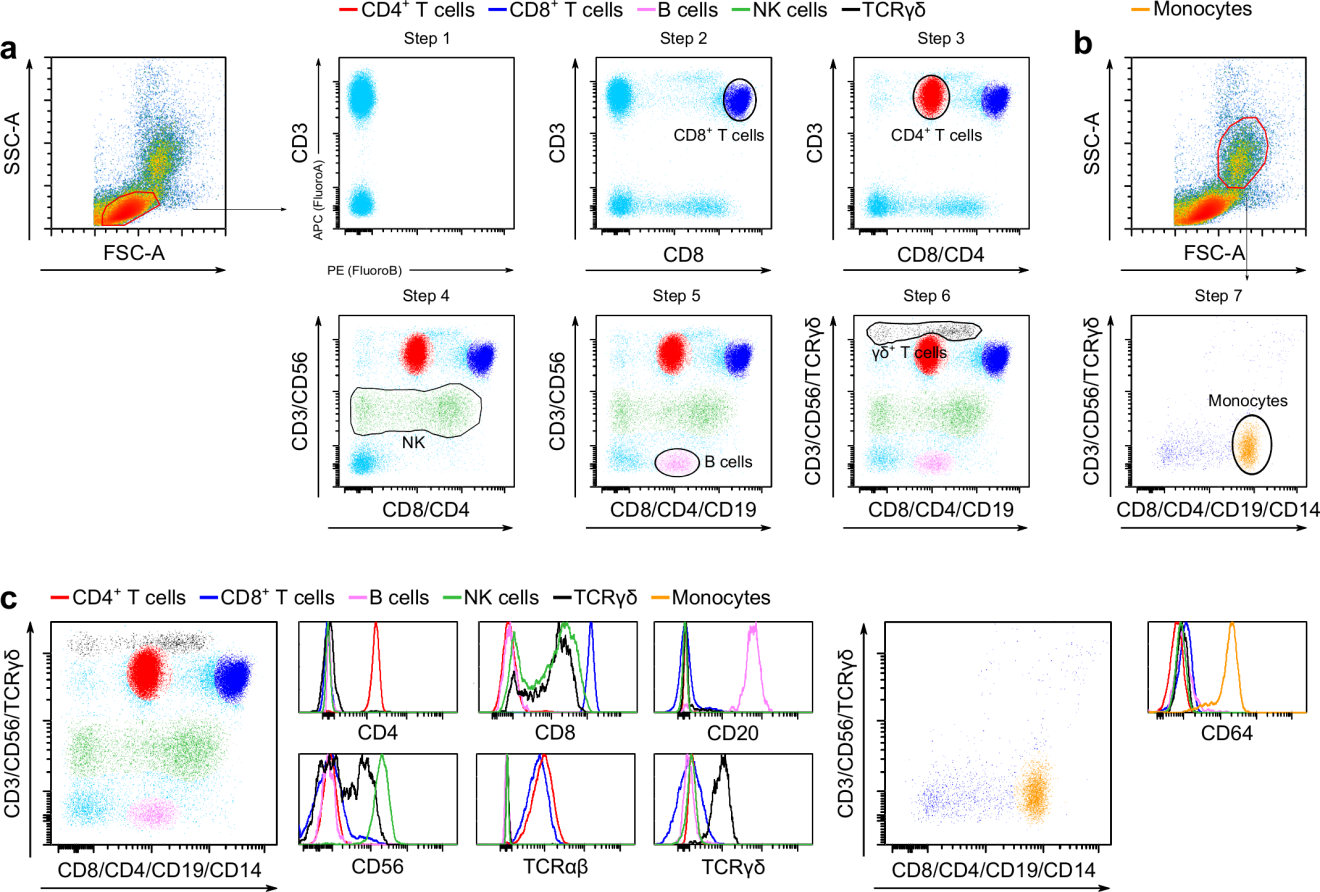
Development and validation of the two-fluorochrome immune-cell staining strategy. PBMCs were isolated from a healthy donor and stained as described. (**a**) Lymphocytes were gated on the basis of their FSC-A and SSC-Area. To develop the final panel six steps were taken to incorporate a marker at the time as described in the text. Step 6 represents the complete array of lymphocyte populations that can be identified with the two-fluorochrome immune-cell staining. (**b**) Monocytes were gated on the basis of their FSC-A and SSC-Area and their flow cytometric profile with the complete two-fluorochrome immune-cell staining is shown. (**c**) PBMC were simultaneously stained with the two-fluorochrome immune-cell panel and with alternative antibody clones or markers conjugated with different fluorochromes directed against the same cell population. This data is representative of ≥ 10 experiments.

To stain seven markers with two fluorochromes (named for simplicity throughout the manuscript respectively fluorochrome A and B), we utilized a step-by-step strategy incorporating one marker at the time. Initial experiments were performed on freshly purified human peripheral blood mononuclear cells (PBMC). Lymphocytes and monocytes were separated using a gating strategy based on both their forward vs. side scatter profile and lineage marker expression (Fig 1A and 1B and S1 Fig), while dead cells were excluded with a live/dead marker. First, extracellular staining with anti-CD3ε (fluorochrome A) was used as surrogate marker to differentiate TCR positive and negative cells. CD4^+^ T cell, CD8^+^ T cell and TCR γδ belong to the group of T cell receptor positive (TCR) cells. B cells, and NK cells belong to the TCR negative group (Fig 1A- Step1). We then added anti-CD8 (fluorochrome B). In a dot plot representation with fluorochrome A plotted on the y axis and fluorochrome B plotted on x axis, CD8^+^ lymphocytes were CD3/CD8 double positive, CD4^+^ lymphocytes were single CD3 positive, B cells and NK cells were double negative for CD3 and CD8^+^, NK CD8^+^ were single positive for CD8 (Fig 1A- Step2). The single positive CD3 population and the CD3/CD8 double positive population were separated by more than two logs allowing the resolution of another population in between. Therefore, we devised a strategy to simultaneously identify CD4^+^ and CD8^+^ T cells within the CD3^+^ population by using anti-CD4 and anti-CD8 antibodies conjugated to the same fluorochrome B. To achieve this result the amount of anti-CD4 antibody was titrated to place the CD3/CD4 double positive population in between the CD3^+^/CD8^-^ and the CD3/CD8 double positive, paying attention not to interfere with CD8 mid positive CD8 T cells and TCR γδ, as well as CD4/CD8 double negative TCR γδ cells (Fig 1A- Step3, S2 Fig). In particular, high concentrations of anti-CD4 antibody result in an overlapping of the CD4^+^ T cell signal with the CD8 dim signal, while low concentrations of anti-CD4 result in a poor separation of CD4^+^ T cells from the CD4/CD8 double negative cells (S2 Fig**)**. Applying the same concept that we used to discriminate CD4^+^ and CD8^+^ T cells, we could differentiate CD56^+^ NK from CD3^+^ cells using an anti-CD56 titrated antibody in fluorochrome A (Fig 1A- Step4). This technique allowed also to identify CD8/CD56 double positive NK cells. B cells that are negative for CD3 were identified by adding an anti-CD19 antibody conjugated with fluorochrome B (Fig 1A- Step5). TCR γδ lymphocytes are a heterogeneous population of mainly CD4/CD8 double negative or CD8 single positive cells. It has been reported that CD3 levels are higher in TCR γδ than in TCR αβ[4], and therefore TCR γδ^+^ lymphocytes tend to segregate from CD4^+^ and CD8^+^ T cells even without using any additional marker. To further improve the separation of this cell population anti-TCR γδ was added in fluorochrome A (Fig 1A- Step6). Classical monocytes, which are negative for CD3, were identified by a forward/side scatter gating strategy and the expression of the specific lineage marker CD14 using an antibody conjugated with fluorochrome B (Fig 1B-Step7). Through this step wise approach, we arrived at a final two-fluorochrome immune-cell profiling panel that uses as markers CD3/CD56/TCR γδ in fluorochrome A and CD4/CD8/CD14/CD19 in fluorochrome B. Utilizing this set of markers, the staining approach was not affected by culturing PBMC overnight at 37°C or cryopreservation and could be applied to whole blood samples (S3 Fig). We also successfully tested different combination of fluorochromes and instruments (S4 Fig). Finally, we tested how this methodology is affected by fluctuations in the cell number by keeping constant the concentration of antibodies while varying the number of cells from 1*10^6^ to 7.5*10^3^ with a two-fold serial dilution. Reducing the number of cells did not impact the separation of the different cell populations. This result is in line with the general concept in flow cytometry that the concentration of antibodies is more important than the number of cells in each sample (S5 Fig).

Together these data demonstrate that our method can be applied to a variety of fresh and stored samples derived from human blood and can be easily adapted to different instrumentation and cell number.

Standard flow cytometry controls, such as isotype and fluorescent-minus-one controls, are not appropriate to detect non-specific signals caused by cross reactivity of multiple antibodies labeled with the same fluorochorome. Thus, we developed an internal control staining method to validate the specificity of the staining for every population. For each marker (e.g CD4), we selected an antibody clone not competing with the clone used in the two-fluorochrome immune-cell profiling procedure (CD4 clone OKT4 as alternative to clone RPA-T4, CD8 clone HIT 8a as alternative to clone RPA-T8, CD56 clone REA-196 as alternative to clone B159) and selected a different fluorochrome. For targets where we couldn’t identify pairs of non-competing commercially available clones, we utilized alternative lineage markers targeting the same population (TCR αβ as alternative to CD3, CD64 as alternative to CD14, CD20 as alternative to CD19). We reasoned that if the staining obtained with our two-fluorochrome approach is specific, one would expect that each population is also positive for its own alternative lineage clone/marker. Therefore, we simultaneously stained freshly isolated PBMC with the two-fluorochrome immune-cell profiling procedure and the seven alternative clones/markers conjugated with different fluorochromes (as reported in the Material and Methods section). As anticipated, αβ T cells (CD3^+^/TCR γδ^-^), γδ T cells (CD3^+^/TCR γδ^+^), CD4^+^ T cells (CD3^+^/CD4^+^), B cells (CD3^-^/CD19^+^), and monocytes (CD3^-^/CD14^+^) uniformly express respectively TCR αβ, TCR γδ, CD4, CD20, and CD64 detected using the alternative reagent (Fig 1C) (www.hcdm.org)[2]. Also, CD8 detected using clone HIT 8a, is uniformly expressed at high levels in CD8^+^ T cells (CD3^+^/CD8^+^), and at lower levels by some NK and γδ T cells (Fig 1C). Likewise, CD56 is expressed by all NK cells (CD3^-^/CD56^+^) and by a portion of γδ T cells, as previously reported (Fig 1C)[5]. As further validation, we stained in parallel PBMCs from six individual donors with a standard seven marker/seven fluorochrome or with our two-fluorochrome immune-cell profiling approach and then compared the percentage of the different subpopulations using nonparametric Wilcoxon signed-ranked test. No statistically significant differences were found among any of the analyzed populations. (S1 Table). These data show that the two-fluorochrome staining approach specifically identify CD4^+^ T cells, CD8^+^ T cells, γδ T cells, B cells, NK cells and classical monocytes with an accuracy comparable to a standard seven marker/seven fluorochrome staining.

**Fig 2:**
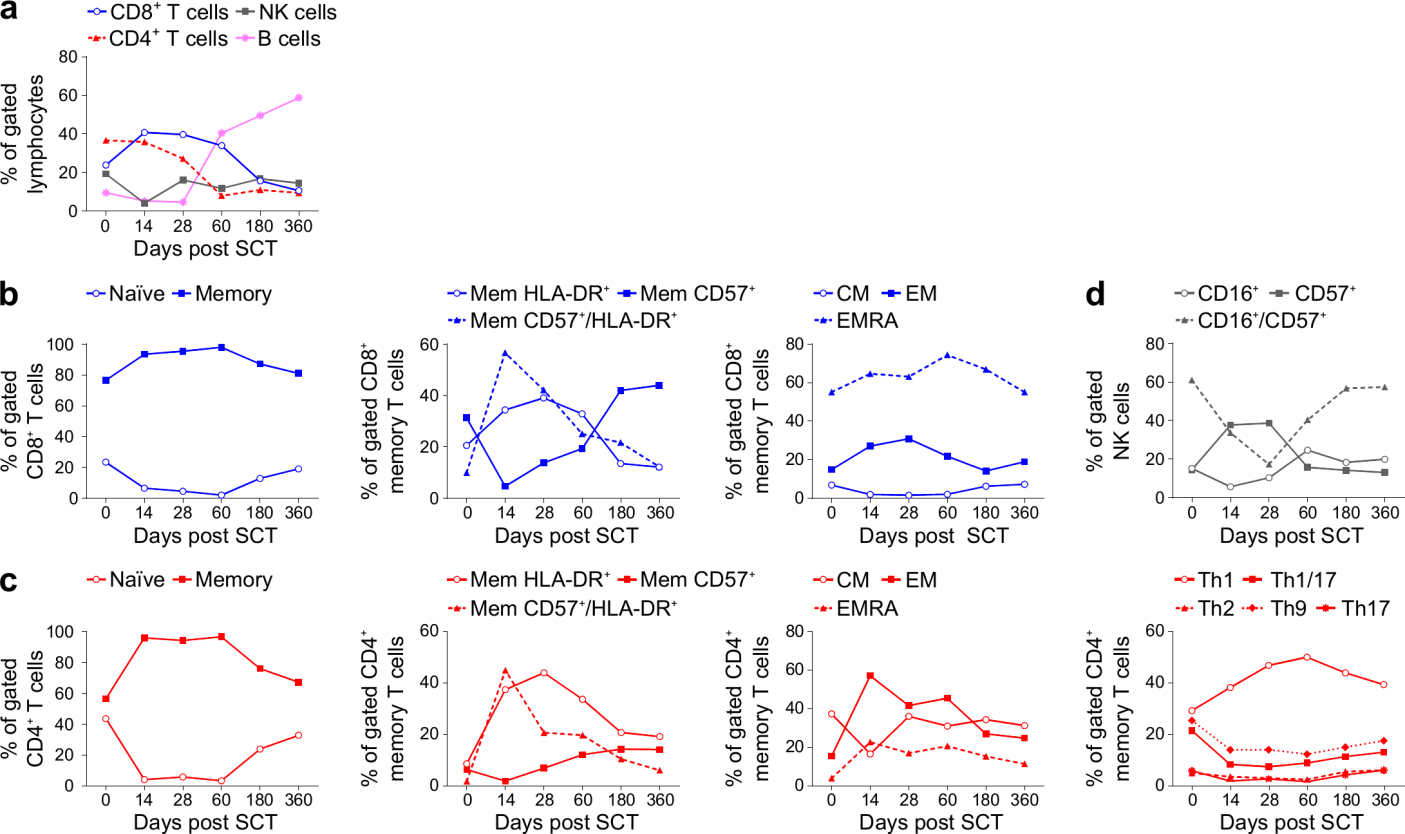
Two-fluorochrome immune-cell staining of cryo-preserved PBMC isolated from patients with multiple myeloma. PBMC isolated from patient with multiple myeloma involved in a clinical trial were collected and viable cryo-preserved at day 0, 14, 28, 60, 180 and 360 after stem cell transplant (SCT). (**a**) Frequency of the major lymphoid populations identified by the two-fluorochrome immune-cell staining. (**b**) Frequency over time of: naïve and memory CD8^+^ T cells; HLA-DR and CD57 memory CD8^+^ T cells; central memory (CM), effector memory (EM) and effector memory CD45RA (EMRA) CD8^+^ T cells. (**c**) Frequency over time of: naïve and memory CD4^+^ T cells; HLA-DR and CD57 memory CD4^+^ T cells; central memory (CM), effector memory (EM) and effector memory CD45RA^+^ (EMRA) CD4^+^ T cells; Th1, Th1/17, Th2, and Th17 CD4^+^ T cells (**d**) NK cell subsets.

Increasing the number of parameters simultaneously analyzed by flow cytometry has been crucial to study with greater precision the function and the kinetics of immune cells in health and disease state. To fully exploit the potential of the technique presented here we developed a staining panel that enabled us to further characterize each lymphocyte population with regards to their naïve/memory profile (CD45RA, CCR7), T effector phenotype (CCR4, CCR6, CXCR3), activation and cell exhaustion status (HLA-DR, CD57, CDRA^+^ effector memory, CD16) (S6 Fig)[6–15]. As proof of principle, we applied this panel to document lymphocyte population dynamics over the course of a year in a patient with multiple myeloma involved in an adoptive T cell therapy trial using cryopreserved samples[3]. Reconstitution of the patient with stem cells and self-derived bone marrow infiltrating lymphocytes resulted in a profound CD4^+^ and CD8^+^ T cell memory activation accompanied by a marked increase in HLA-DR and CD57 expression, whereas the naïve population was reduced dramatically. The T helper response was skewed towards a Th1 phenotype with no changes in the Th2 and Th17 subsets. At day 60 the percentage of B cells dramatically increased preceding the patient relapse (Fig 2 and S7 Fig). Altogether, these data show that the proposed immunophenotyping strategy is applicable to samples collected over a prolonged period of time and can effectively detect very different lymphocyte dynamics.

## Discussion

We have developed a comprehensive easy-to-apply methodology that can be integrated in current staining protocols to generate a footprint of the major human immune populations by using only two fluorochromes. Other reports have successfully combined few markers in two fluorochrome to positively identify and separate immune cell populations. [16–18]. Combining multiple antibodies in single channels is an accepted methodology that has also been implemented in clinical standardized protocols to identify malignant leukocytes [19]. The combination of seven markers in two fluorochromes has already been attempted by Bradford et al. [20] Their technique has some limitation in that, despite using different cell markers, it relies on a complex in-house individually labeled antibody with different amount of fluorochromes. In contrast, our approach is simply based on titration of commercially available antibodies, as required by any staining protocol. In particular, CD3, CD8, CD19, CD14 and TCR γδ antibodies are titrated using the maximum stain index or a saturation curve, whereas only CD4 and CD56 require a specific titration, as described before. The anti-CD4 antibody should be titrated to separate CD4^+^ T cells from CD8 dim populations allowing to identify CD8 TCR γδ cells and dim CD8 T cells. This is important in diseases conditions, such as HIV-1 infection, where dim CD8 T cells are greatly expanded. Of note, the lower expression of CD56 in NK cells compared to CD3 in T lymphocytes greatly facilitates the titration of the anti CD56 antibody [21]. Therefore, the protocol presented here can be adapted to use antibodies from different vendors or different fluorochromes solely by titrating the antibodies, which is always required when antibodies and fluorochromes are selected for a new staining panel. To achieve an optimal separation of all the subpopulation, high quantum yield fluorochromes, such as APC, BV421 and PE, should be chosen.

The two-fluorochrome immune-cell staining protocol minimizes the number of fluorochromes used to classify key immune cell subsets providing space in the staining panel for five additional markers than can be simultaneously acquired on most flow cytometers to interrogate complex cell populations. This is especially important now that a new generation of affordable 6–10 fluorochrome flow cytometers is making flow cytometry accessible to most laboratories. Two-tree fluorochrome instruments developed for field service in remote areas can also adopt this approach to easily enumerate several cell populations and improve the output of research with limited equipment and resources. On the other hand, more advanced instruments can benefit from having extra fluorochromes available to achieve a deeper flow cytometry analysis while reducing the requirement of skilled operators to deal with spillover of fluorochromes in different detectors. Selecting fluorochromes with little fluorescent signal spillover (e.g. PE and APC, or PE and BV421) minimizes compensation issues and reduces the complexity of the experiment.

We acknowledge that a single fluorochrome-single marker strategy is still the most accurate approach to identify different immune populations and discriminate rare cell populations, such as NKT cells. Nevertheless, even if NKT cells cannot be properly gated with a two-fluorochrome staining protocol, it should be emphasized that in our tests the separation between CD4 and CD8 is sufficient to exclude CD8 dim populations (NKT) from the CD4 gate, as demonstrated by the absence of CD8 expression in the CD4 validation gate, and CD56 in the CD8 gate (Fig 1C). Of note, the same kind of separation problem between CD8 and NKT cells is present in the widely adopted staining strategy used to identify CD4 and CD8 T lymphocytes which is based on antibodies against CD3, CD4 and CD8. Despite limited resolution of specific cell populations, we successfully investigated clinical samples from patients with multiple myeloma and from patients with systemic sclerosis or Lyme disease showing that this staining procedure was not influenced by drug treatment, chronic immune activation or ongoing infectious diseases and can therefore be successfully adopted to study longitudinal clinical samples. It remains important that for each specific study preliminary testing is conducted to assess the feasibility and accuracy of this proposed protocol, because there could be instances, such us viral infections or chronic inflammations, in which immune cells are characterized by aberrant expression of lineage markers that might preclude our approach to work.

The approach we describe is highly flexible and can be scaled down using different combination of markers to target only specific cell populations. As an example, if there is no interest in characterizing B cells, CD19 can be omitted, or conversely if B cells and NK are the sole focus of the analysis, it is sufficient to use CD19, CD56 and CD8. In addition, if some of the detectors of the instrument are not used during the analysis, they could be used to move some markers of the two-fluorochrome staining to a different detector/fluorochrome to better identify rare cell populations (i.e. NKT cells or non-classical monocytes).

In conclusion, we present a method to expand the number of parameters recordable on flow cytometers allowing for a more exhaustive study of the repertoire of immune cells that can be characterized in a quick, robust and cost-efficient manner. Similar approaches, aiming at expanding the number of recordable markers, may also be developed for different type of animal models or other applications beyond human immune cell profiling.

## Supporting information

S1 Fig Gating strategy of the two-fluorochrome immune-cell staining. Live cells were defined by FSC-A and SSC-Area. FSC-Height vs FSC-Width and SSC-Height vs SSC-Width were used to exclude cell aggregates from the analysis. Live/dead fixable blue dead cell stain was used to remove dead cells and red blood cells that may be present in PBMC preparation. Lymphocytes and monocytes were discriminated by FSC-A and SSC-Area.
(TIF)

S2 Fig Two-fluorochrome immune-cell staining testing different concentrations of anti CD4 antibody. Representative staining of PBMC isolated and stained with decreasing concentrations of BV421 anti-CD4 antibody as indicated. Cells were also stained with the other markers of the two-fluorochrome immune-cell panel at the standard concentration. Analysis was done on gated CD3 positive cells. Color code of the concentrations: green indicates concentrations that result in an optimal separation of CD4^+^ T cells from the other CD3^+^ populations; orange indicates concentrations that result in an acceptable but not ideal separation; red indicates concentrations that result in poor separation of CD4^+^ T cells from dim CD8 cells or CD4/CD8 double negative populations.
(TIFF)

S3 Fig Two-fluorochrome immune-cell staining of different blood derived samples. Panels depict lymphocytes (left) and monocytes (right) analyzed with the two-fluorochrome immune-cell staining performed on samples (**a**) kept in culture over night at 37°C, (**b**) cryo-preserved, or (**c**) on whole blood.
(TIFF)

S4 Fig Two-fluorochrome immune-cell staining strategy using different fluorochromes or cytometer. PBMC were isolated from healthy donor, patients with systemic sclerosis and Lyme disease and stained as described. (**a**) Lymphocytes were gated on the basis of their FSC-A and SSC-Area. To develop the final panel six steps were taken to incorporate a marker at a time using the fluorochromes BV421 and PE. Step 6 represents the complete array of lymphocyte populations that can be identified with the two-fluorochrome immune-cell staining. (**b**) Monocytes were gated on the basis of their FSC-A and SSC-Area and their flow cytometric profile with the complete two-fluorochrome (BV421 and PE) immune-cell staining is shown. (**c**) Panels depict lymphocytes (left) and monocytes (right) analyzed with the two-fluorochrome immune-cell staining performed on FACSCanto flow cytometer.
(TIFF)

S5 Fig Two-fluorochrome immune-cell staining strategy using different number of cells. Different number of PBMC isolated from healthy donors, as indicated above each plot, were stained with the two-fluorochrome immune-cell method. (**a**) Lymphocytes were gated on the basis of their FSC-A and SSC-Area. A representative plot with the gating strategy used to identify the main immune populations has been included. (**b**) Monocytes were gated on the basis of their FSC-A and SSC-Area. A representative plot with the gating strategy used to identify the main immune populations has been included. (**c**) Percentages of cell populations were compared for different number of cells.
(TIFF)

S6 Fig Gating strategy used to analyze samples of PBMC isolated from a patient with multiple myeloma. Representative analysis at day 0 after stem cell transplant (SCT). (**a**) Lymphocytes were gated on the basis of their FSC-A and SSC-Area and their flow cytometric profile with the two-fluorochrome immune-cell staining is shown. (**b**) B cells of some patients with multiple myeloma have been reported to express the NK marker CD56. To exclude any possible contamination of B cells in the NK population we first gated on the NK and B cell population, and then identified B cells and NK cells based on their distinct expression of HLA-DR and CCR6. (**c**) CD45RA and CCR7 were used to identify naïve (CD45RA^+^/CCR7^+^), central memory (CM, CD45RA^-^/CCR7^+^), effector memory (EM, CD45RA^-^/CCR7^-^) and effector memory CD45RA^+^ (EMRA, CD45RA^+^/CCR7^+^) CD8^+^ T cells. (**d**) HLA-DR and CD57 expression in CD8^+^ naïve and memory population (which comprise CM, EM and EMRA), CM, EM and EMRA. (**e**) CD45RA and CCR7 were used to identify naïve (CD45RA^+^/CCR7^+^), central memory (CM, CD45RA^-^/CCR7^+^), effector memory (EM, CD45RA^-^/CCR7^-^) and effector memory CD45RA^+^ (EMRA, CD45RA^+^/CCR7^+^) CD4^+^ T cells. (**f**) HLA-DR and CD57 expression in CD8^+^ naïve and memory population (which comprise CM, EM and EMRA), CM, EM and EMRA. (**g**) CCR4 and CCR6 were used as marker to identify within the memory population Th9 CD4^+^ T cells (**h**) CCR4, CCR6 and CXCR3 were used as marker to identify within the memory population Th1, Th1/17, Th2 and Th17 CD4^+^ T helper subpopulations. (**i**) CD16 and CD57 expression in NK cells.
(TIFF)

S7 Fig Two-fluorochrome immune-cell staining of cryo-preserved PBMC isolated from patients with multiple myeloma. PBMC isolated from a patient with multiple myeloma involved in a clinical trial were collected and viable cryo-preserved at day 0, 14, 28, 60, 180 and 360 after stem cell transplant (SCT). Frozen cells from all the time points were thawed, stained and analyzed by flow cytometry on the same day. A panel of markers was developed to simultaneously stain PBMC with the two-fluorochrome immune-cell and a panel of markers to further characterize each lymphocyte population focusing on CD4^+^ and CD8^+^ naïve/memory T cells, CD4^+^ T helper subpopulations, and activation and cell exhaustion status of CD4^+^, CD8^+^ and NK cells. (**a**) HLA-DR and CD57 expression in central memory (CM), effector memory (EM) and effector memory CD45RA^+^ (EMRA) CD8^+^ T cells. (**b**) HLA-DR and CD57 expression in central memory (CM), effector memory (EM) and effector memory CD45RA^+^ (EMRA) CD4^+^ T cells.
(TIFF)

S1 Table Comparison of the two-fluorochrome immune-cell staining and classical staining approaches. PBMC from six donors were stained with either the two-fluorochrome immune-cell staining (2-Fluoro) or a classical seven-fluorochrome staining (Classic), and the percentages of different populations were compared. “Lymphocytes” is the parent gate of CD3^+^ and CD3^-^. CD3^+^ is the parent gate of gates CD3^+^/CD4^+^, CD3^+^/CD8^+^and CD3^+^/TCR γδ^+^, while CD3^-^ is the parent gate for gates CD3^-^/CD19^+^, CD3^-^/CD56^+^ and CD56^+^CD8^+^. “Monocytes” is the parent gate of CD14^+^ cells.
(DOCX)

S1 Material and Methods List of antibodies and clones used to stain samples. (DOCX)
