# Supplementary figures and images for: Flow cytometric discrimination of seven lineage markers by using two fluorochromes
Source: PLoS One. 2017 Nov 30;12(11):e0188916. doi: 10.1371/journal.pone.0188916 (PMC5708788; doi:10.1371/journal.pone.0188916)

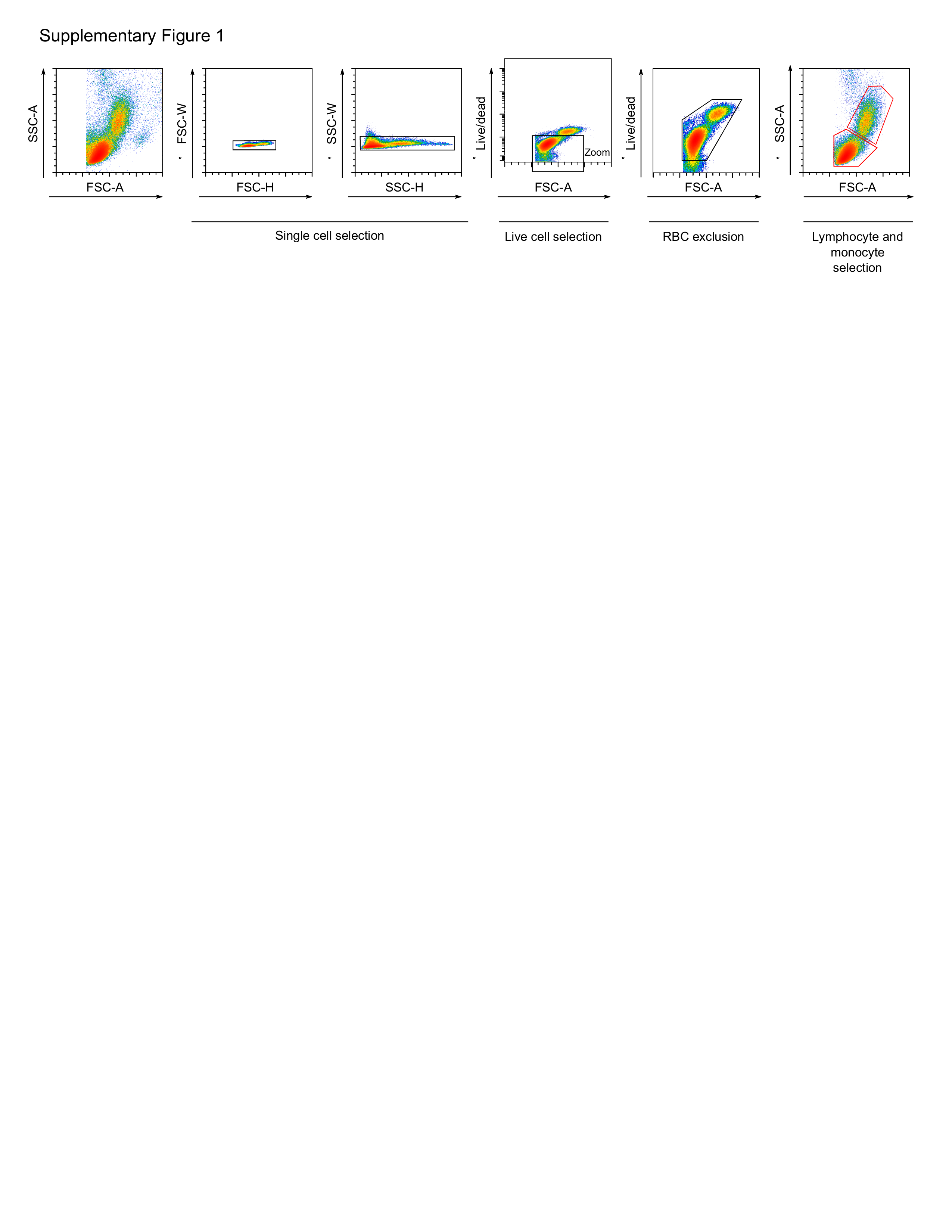

Supplement: S1 Fig — Live cells were defined by FSC-A and SSC-Area. FSC-Height vs FSC-Width and SSC-Height vs SSC-Width were used to exclude cell aggregates from the analysis. Live/dead fixable blue dead cell stain was used to remove dead cells and red blood cells that may be present in PBMC preparation. Lymphocytes and monocytes were discriminated by FSC-A and SSC-Area. (TIF) [file pone.0188916.s001.tif]

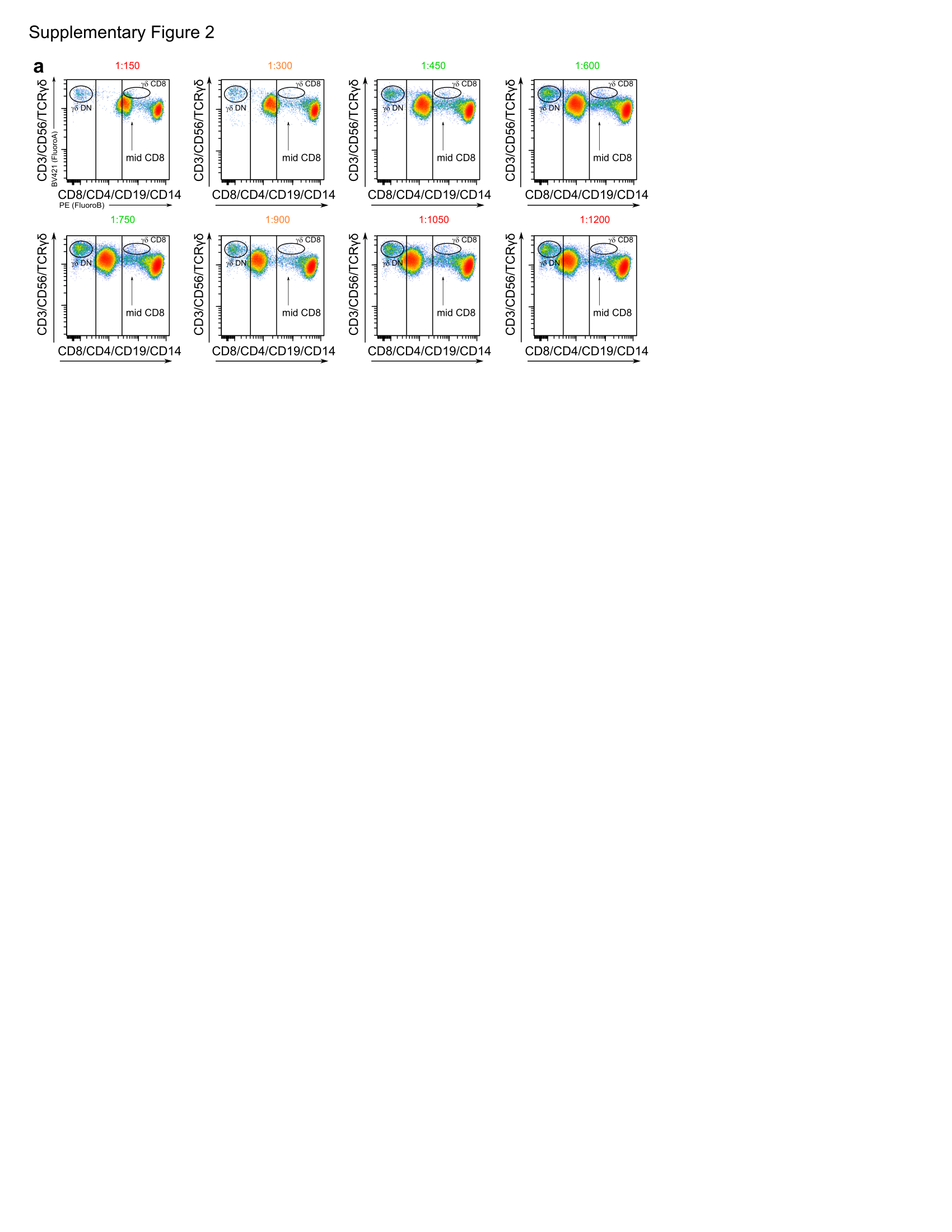

Supplement: S2 Fig — Representative staining of PBMC isolated and stained with decreasing concentrations of BV421 anti-CD4 antibody as indicated. Cells were also stained with the other markers of the two-fluorochrome immune-cell panel at the standard concentration. Analysis was done on gated CD3 positive cells. Color code of the concentrations: green indicates concentrations that result in an optimal separation of CD4+ T cells from the other CD3+ populations; orange indicates concentrations that result in an acceptable but not ideal separation; red indicates concentrations that result in poor separation of CD4+ T cells from dim CD8 cells or CD4/CD8 double negative populations. (TIFF) [file pone.0188916.s002.tiff]

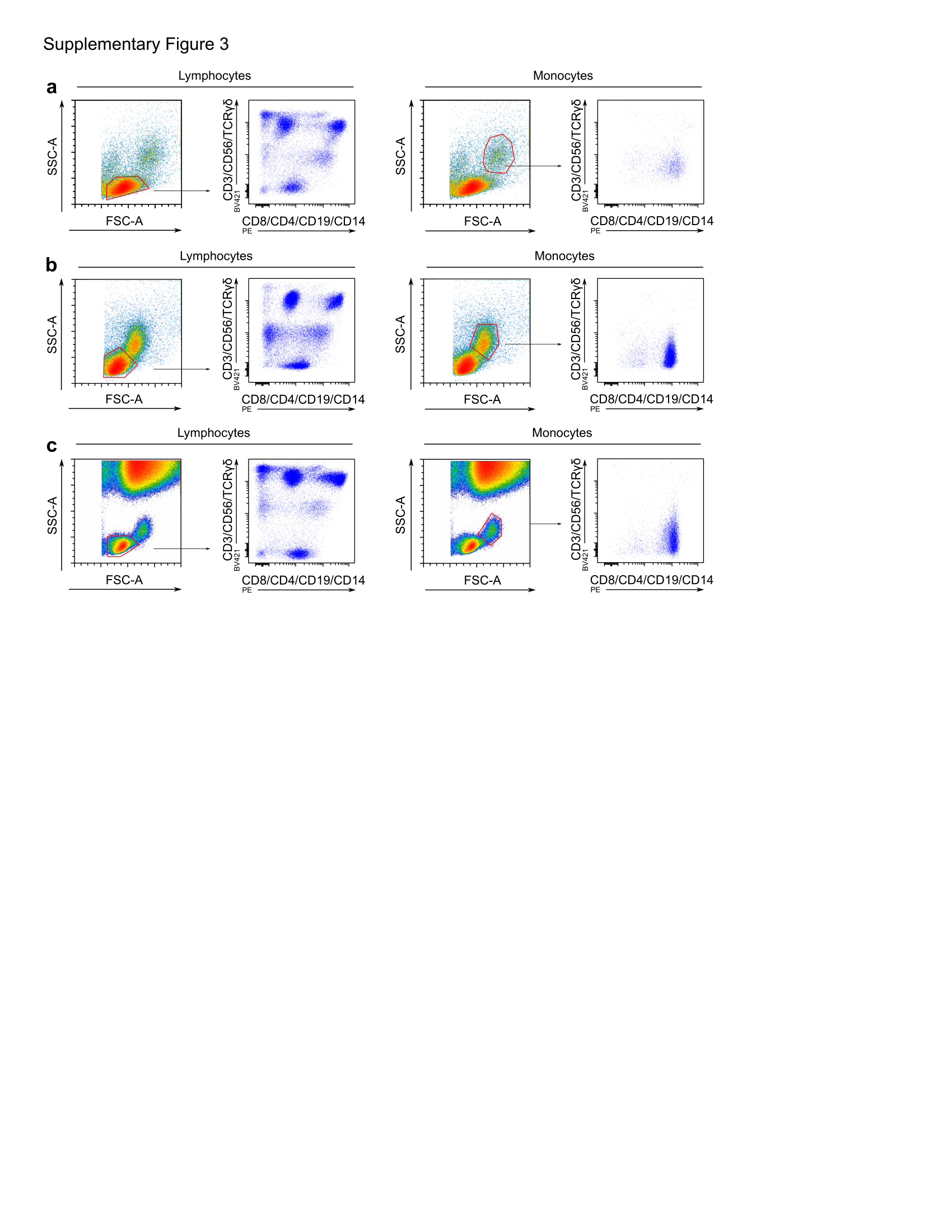

Supplement: S3 Fig — Panels depict lymphocytes (left) and monocytes (right) analyzed with the two-fluorochrome immune-cell staining performed on samples (a) kept in culture over night at 37°C, (b) cryo-preserved, or (c) on whole blood. (TIFF) [file pone.0188916.s003.tiff]

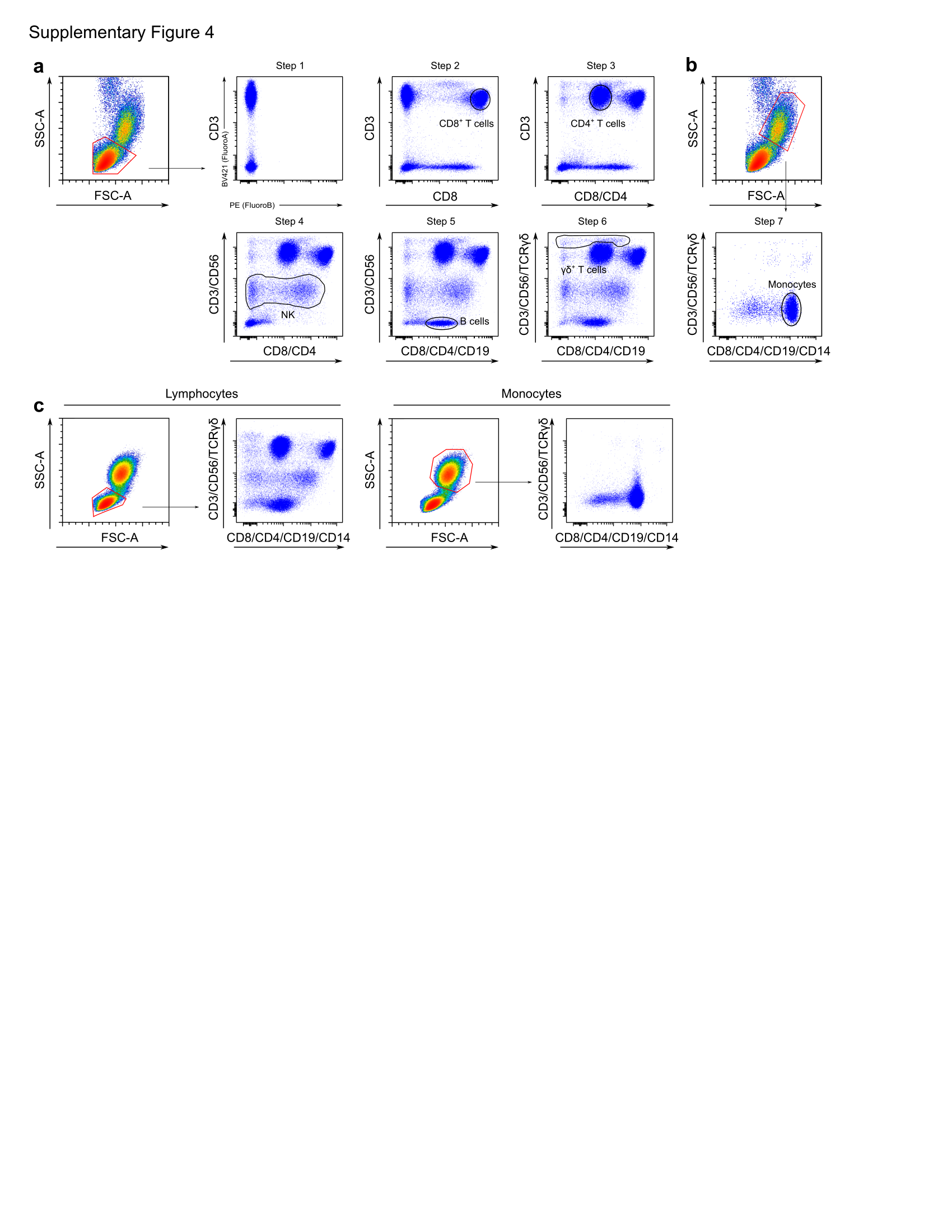

Supplement: S4 Fig — PBMC were isolated from healthy donor, patients with systemic sclerosis and Lyme disease and stained as described. (a) Lymphocytes were gated on the basis of their FSC-A and SSC-Area. To develop the final panel six steps were taken to incorporate a marker at a time using the fluorochromes BV421 and PE. Step 6 represents the complete array of lymphocyte populations that can be identified with the two-fluorochrome immune-cell staining. (b) Monocytes were gated on the basis of their FSC-A and SSC-Area and their flow cytometric profile with the complete two-fluorochrome (BV421 and PE) immune-cell staining is shown. (c) Panels depict lymphocytes (left) and monocytes (right) analyzed with the two-fluorochrome immune-cell staining performed on FACSCanto flow cytometer. (TIFF) [file pone.0188916.s004.tiff]

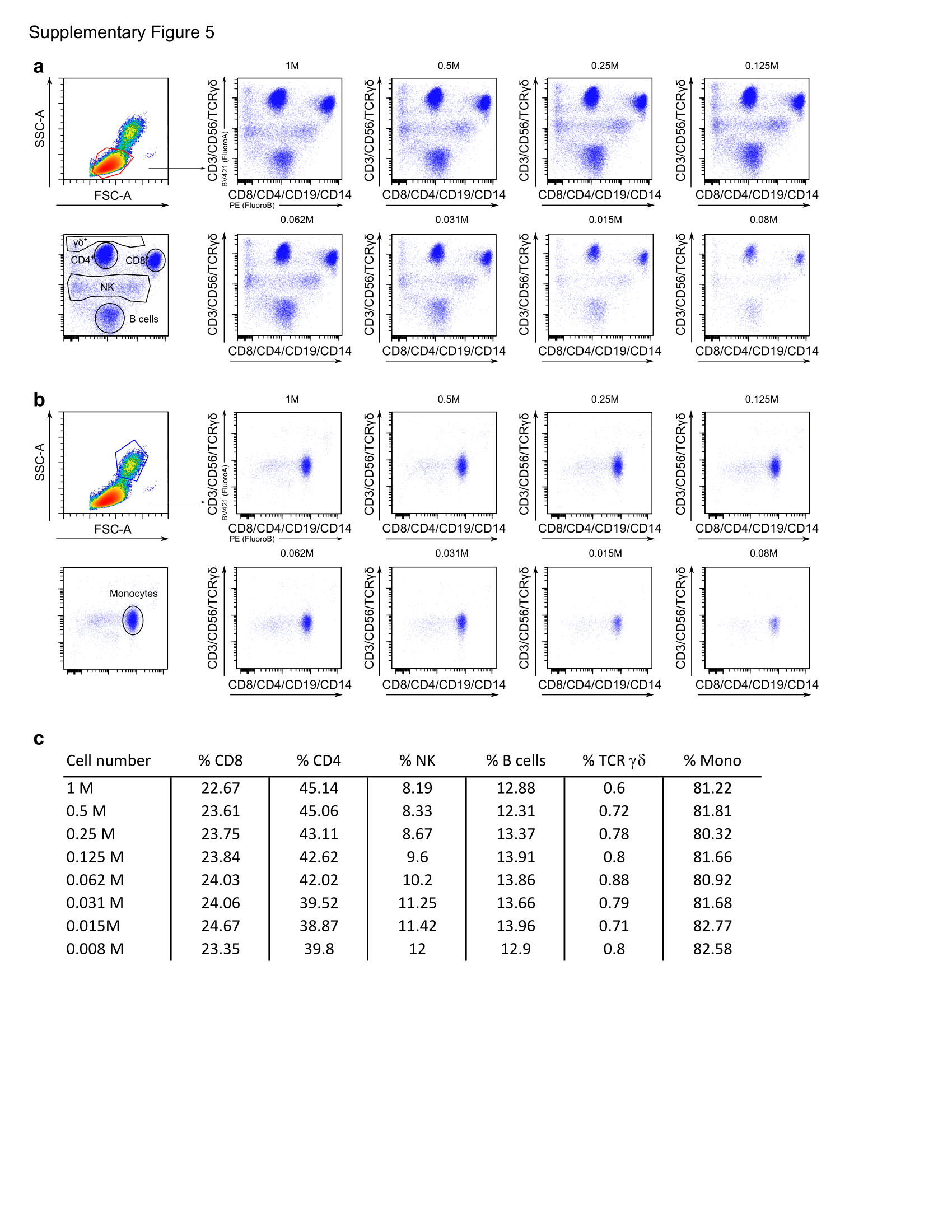

Supplement: S5 Fig — Different number of PBMC isolated from healthy donors, as indicated above each plot, were stained with the two-fluorochrome immune-cell method. (a) Lymphocytes were gated on the basis of their FSC-A and SSC-Area. A representative plot with the gating strategy used to identify the main immune populations has been included. (b) Monocytes were gated on the basis of their FSC-A and SSC-Area. A representative plot with the gating strategy used to identify the main immune populations has been included. (c) Percentages of cell populations were compared for different number of cells. (TIFF) [file pone.0188916.s005.tiff]

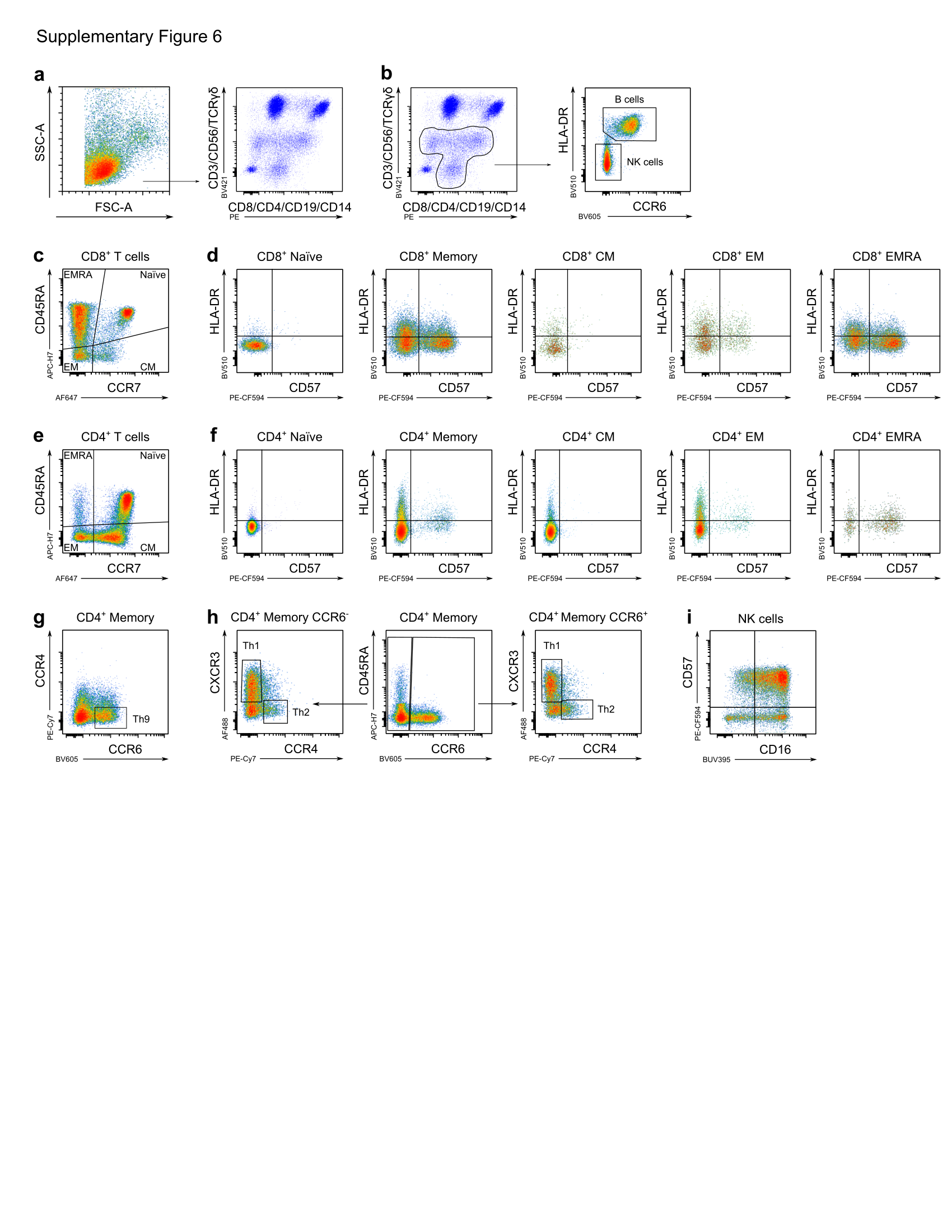

Supplement: S6 Fig — Representative analysis at day 0 after stem cell transplant (SCT). (a) Lymphocytes were gated on the basis of their FSC-A and SSC-Area and their flow cytometric profile with the two-fluorochrome immune-cell staining is shown. (b) B cells of some patients with multiple myeloma have been reported to express the NK marker CD56. To exclude any possible contamination of B cells in the NK population we first gated on the NK and B cell population, and then identified B cells and NK cells based on their distinct expression of HLA-DR and CCR6. (c) CD45RA and CCR7 were used to identify naïve (CD45RA+/CCR7+), central memory (CM, CD45RA-/CCR7+), effector memory (EM, CD45RA-/CCR7-) and effector memory CD45RA+ (EMRA, CD45RA+/CCR7+) CD8+ T cells. (d) HLA-DR and CD57 expression in CD8+ naïve and memory population (which comprise CM, EM and EMRA), CM, EM and EMRA. (e) CD45RA and CCR7 were used to identify naïve (CD45RA+/CCR7+), central memory (CM, CD45RA-/CCR7+), effector memory (EM, CD45RA-/CCR7-) and effector memory CD45RA+ (EMRA, CD45RA+/CCR7+) CD4+ T cells. (f) HLA-DR and CD57 expression in CD8+ naïve and memory population (which comprise CM, EM and EMRA), CM, EM and EMRA. (g) CCR4 and CCR6 were used as marker to identify within the memory population Th9 CD4+ T cells (h) CCR4, CCR6 and CXCR3 were used as marker to identify within the memory population Th1, Th1/17, Th2 and Th17 CD4+ T helper subpopulations. (i) CD16 and CD57 expression in NK cells. (TIFF) [file pone.0188916.s006.tiff]

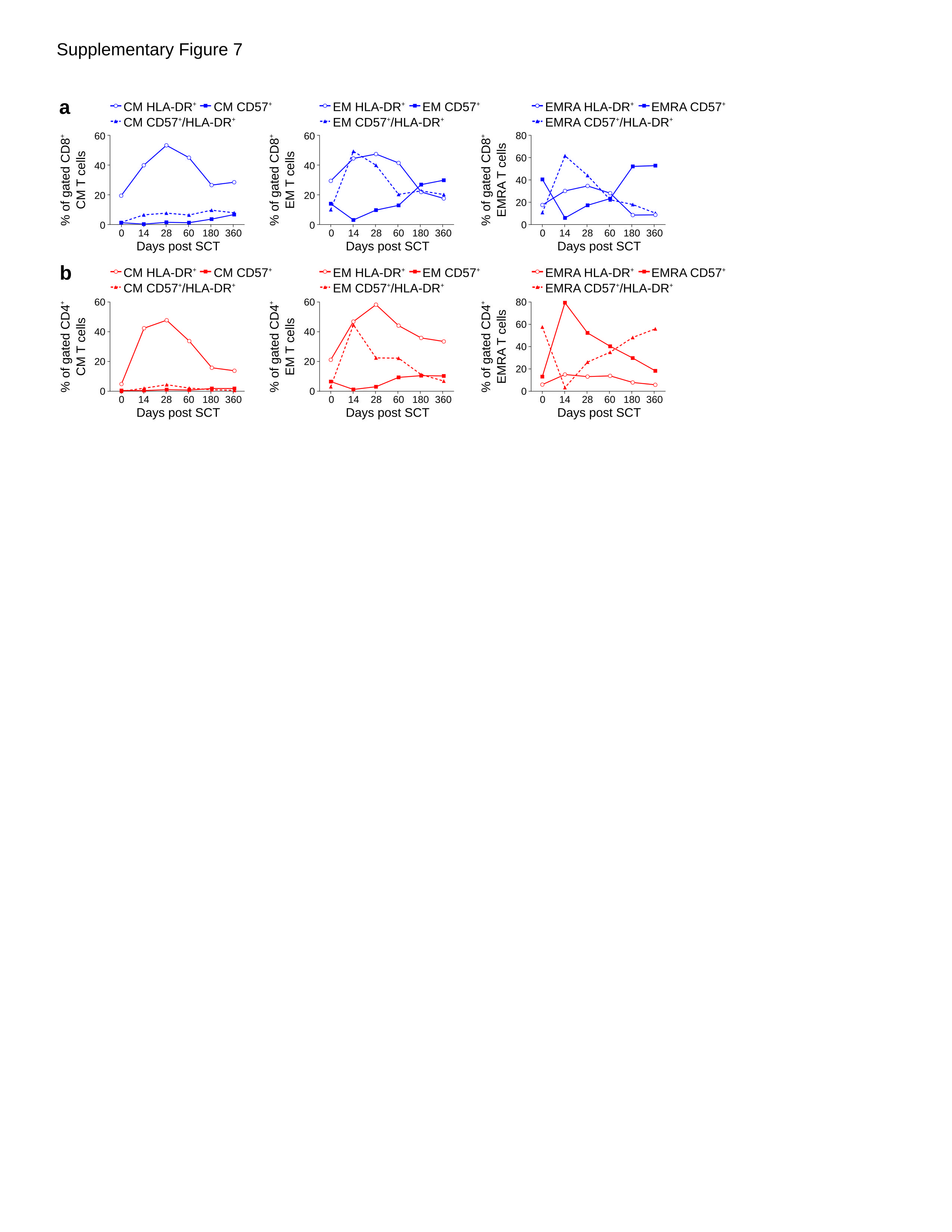

Supplement: S7 Fig — PBMC isolated from a patient with multiple myeloma involved in a clinical trial were collected and viable cryo-preserved at day 0, 14, 28, 60, 180 and 360 after stem cell transplant (SCT). Frozen cells from all the time points were thawed, stained and analyzed by flow cytometry on the same day. A panel of markers was developed to simultaneously stain PBMC with the two-fluorochrome immune-cell and a panel of markers to further characterize each lymphocyte population focusing on CD4+ and CD8+ naïve/memory T cells, CD4+ T helper subpopulations, and activation and cell exhaustion status of CD4+, CD8+ and NK cells. (a) HLA-DR and CD57 expression in central memory (CM), effector memory (EM) and effector memory CD45RA+ (EMRA) CD8+ T cells. (b) HLA-DR and CD57 expression in central memory (CM), effector memory (EM) and effector memory CD45RA+ (EMRA) CD4+ T cells. (TIFF) [file pone.0188916.s007.tiff]
