# Supplementary material for: Flow cytometric discrimination of seven lineage markers by using two fluorochromes
Source: PLoS One. 2017 Nov 30;12(11):e0188916. doi: 10.1371/journal.pone.0188916 (PMC5708788; doi:10.1371/journal.pone.0188916)
Supplement: S1 Table — PBMC from six donors were stained with either the two-fluorochrome immune-cell staining (2-Fluoro) or a classical seven-fluorochrome staining (Classic), and the percentages of different populations were compared. “Lymphocytes” is the parent gate of CD3+ and CD3-. CD3+ is the parent gate of gates CD3+/CD4+, CD3+/CD8+and CD3+/TCR γδ+, while CD3- is the parent gate for gates CD3-/CD19+, CD3-/CD56+ and CD56+CD8+. “Monocytes” is the parent gate of CD14+ cells. (DOCX) [file pone.0188916.s008.docx]

**Supplementary Table 1** | Comparison of the two-fluorochrome immune-cell staining and classical staining approaches.

|  | Donor 1 | |  | Donor 2 | |  | Donor 3 | |
| --- | --- | --- | --- | --- | --- | --- | --- | --- |
|  | 2-Fluoro | Classic |  | 2-Fluoro | Classic |  | 2-Fluoro | Classic |
| Lymphocytes | 86.25% | 87.42% |  | 75.99% | 70.96% |  | 80.40% | 79.04% |
| CD3^+^ | 73.60% | 74.11% |  | 78.20% | 78.47% |  | 65.04% | 61.70% |
| CD3^+^/CD4^+^ | 68.49% | 68.67% |  | 46.21% | 49.80% |  | 61.95% | 61.55% |
| CD3^+^/CD8^+^ | 21.21% | 20.44% |  | 34.42% | 26.48% |  | 28.53% | 27.06% |
| CD3^+^/TCRγδ^+^ | 7.02% | 8.98% |  | 13.92% | 14.14% |  | 0.09% | 0.20% |
|  |  |  |  |  |  |  |  |  |
| CD3^-^ | 25.85% | 25.03% |  | 21.28% | 21.46% |  | 34.62% | 35.53% |
| CD3^-^/CD19^+^ | 40.40% | 42.38% |  | 54.42% | 54.20% |  | 18.89% | 15.90% |
| CD3^-^/CD56^+^ | 40.63% | 39.17% |  | 35.54% | 36.81% |  | 71.91% | 75.16% |
| CD56^+^/CD8^+^ | 58.51% | 57.98% |  | 62.34% | 63.10% |  | 61.26% | 59.19% |
|  |  |  |  |  |  |  |  |  |
| Monocytes | 6.87% | 6.23% |  | 8.28% | 9.68% |  | 5.04% | 5.26% |
| CD14^+^ | 79.79% | 72.40% |  | 71.10% | 73.90% |  | 51.35% | 52.58% |

|  | Donor 4 | |  | Donor 5 | |  | Donor 6 | |
| --- | --- | --- | --- | --- | --- | --- | --- | --- |
|  | 2-Fluoro | Classic |  | 2-Fluoro | Classic |  | 2-Fluoro | Classic |
| Lymphocytes | 84.61% | 84.43% |  | 90.38% | 90.73% |  | 59.64% | 60.62% |
| CD3^+^ | 72.71% | 71.98% |  | 84.76% | 84.01% |  | 66.03% | 65.5% |
| CD3^+^/CD4^+^ | 63.83% | 66.63% |  | 87.37% | 90.38% |  | 95.04% | 93.48% |
| CD3^+^/CD8^+^ | 32.03% | 29.44% |  | 10.61% | 8.71% |  | 3.36% | 4.81% |
| CD3^+^/TCRγδ^+^ | 1.43% | 1.71% |  | 0.17% | 0.07% |  | 0.34% | 0.26% |
|  |  |  |  |  |  |  |  |  |
| CD3^-^ | 28.4% | 27.92% |  | 15.24% | 15.53% |  | 33.97% | 34.15% |
| CD3^-^/CD19^+^ | 55.73% | 56.83% |  | 42.14% | 42.94% |  | 44.38% | 43.59% |
| CD3^-^/CD56^+^ | 33.22% | 34.08% |  | 43.78% | 42.94% |  | 46.72% | 43.66% |
| CD56^+^/CD8^+^ | 57.7% | 57.98% |  | 51.66% | 50.13% |  | 48.32% | 51.53% |
|  |  |  |  |  |  |  |  |  |
| Monocytes | 5.89% | 5.21% |  | 4.36% | 3.5% |  | 23.41% | 17.56% |
| CD14^+^ | 82.2% | 81.55% |  | 85.71% | 82.92% |  | 84.34% | 81.63% |

PBMC from six donors were stained with either the two-fluorochrome immune-cell staining (2-Fluoro) or a classical seven-fluorochrome staining (Classic), and the percentages of different populations were compared. “Lymphocytes” is the parent gate of CD3^+^ and CD3^-^. CD3^+^ is the parent gate of gates CD3^+^/CD4^+^, CD3^+^/CD8^+^and CD3^+^/TCR γδ^+^, while CD3^-^ is the parent gate for gates CD3^-^/CD19^+^, CD3^-^/CD56^+^ and CD56^=^/CD8^+^. “Monocytes” is the parent gate of CD14^+^ cells.
