# Supplementary material for: Flow cytometric discrimination of seven lineage markers by using two fluorochromes
Source: PLoS One. 2017 Nov 30;12(11):e0188916. doi: 10.1371/journal.pone.0188916 (PMC5708788; doi:10.1371/journal.pone.0188916)
Supplement: S1 Material and Methods — (DOCX) [file pone.0188916.s009.docx]

**List of Antibodies**

Antibody concentration can vary depending on the manufacturer and lot number. Therefore, preliminary tests should be done to achieve the optimal signal.

Antibody panel used for the two-fluorochrome immune-cell staining of PBMC (BV421-PE combination).

| Target | Clone | Fluorochrome | Catalog | Vendor | Concentration | Purpose |
| --- | --- | --- | --- | --- | --- | --- |
| CD3 | UCHT1 | BV421 | 562426 | BD | 1/20 | Lineage |
| CD56 | NCAM16.2 | BV421 | 562751 | BD | 1/900 |  |
| TCRγδ | B1 | BV421 | 331217 | Bio | 1/30 |  |
| CD4 | RPA-T4 | PE | 555347 | BD | 1/450 |  |
| CD8 | RPA-T8 | PE | 555367 | BD | 1/20 |  |
| CD14 | M5E2 | PE | 555398 | BD | 1/15 |  |
| CD19 | HIB19 | PE | 555413 | BD | 1/300 |  |
|  |  |  |  |  |  |  |
| Dead cells |  | L/D Blue | L-23105 | LT | 1/300 | Live/Dead discrimination |

BD = BD Biosciences, Bio = BioLegend, eBio = eBioscience, Milt = Miltenyi, LT = Life Technologies

Antibody panel used for the two-fluorochrome immune-cell staining of PBMC (APC-PE combination).

| Target | Clone | Fluorochrome | Catalog | Vendor | Concentration | Purpose |
| --- | --- | --- | --- | --- | --- | --- |
| CD3 | UCHT1 | APC | 555335 | BD | 1/20 | Lineage |
| CD56 | NCAM16.2 | APC | 555518 | BD | 1/60 |  |
| TCRγδ | B1 | APC | 331211 | Bio | 1/30 |  |
| CD4 | RPA-T4 | PE | 555347 | BD | 1/450 |  |
| CD8 | RPA-T8 | PE | 555367 | BD | 1/20 |  |
| CD14 | M5E2 | PE | 555398 | BD | 1/15 |  |
| CD19 | HIB19 | PE | 555413 | BD | 1/300 |  |
|  |  |  |  |  |  |  |
| Dead cells |  | L/D Blue | L-23105 | LT | 1/300 | Live/Dead discrimination |

BD = BD Biosciences, Bio = BioLegend, eBio = eBioscience, Milt = Miltenyi, LT = Life Technologies

Antibody panel used for the two-fluorochrome immune-cell staining of whole blood (BV421-PE combination).

| Target | Clone | Fluorochrome | Catalog | Vendor | Concentration | Purpose |
| --- | --- | --- | --- | --- | --- | --- |
| CD3 | UCHT1 | BV421 | 562426 | BD | 1/80 | Lineage |
| CD56 | NCAM16.2 | BV421 | 562751 | BD | 1/400 |  |
| TCRγδ | B1 | BV421 | 331217 | Bio | 1/200 |  |
| CD4 | RPA-T4 | PE | 555347 | BD | 1/1200 |  |
| CD8 | RPA-T8 | PE | 555367 | BD | 1/100 |  |
| CD14 | M5E2 | PE | 555398 | BD | 1/80 |  |
| CD19 | HIB19 | PE | 555413 | BD | 1/300 |  |

BD = BD Biosciences, Bio = BioLegend, eBio = eBioscience, Milt = Miltenyi, LT = Life Technologies

Antibody panel used to discriminate seven lineage markers using two fluorochromes and to internally validate the accuracy of the proposed staining methodology.

| Target | Clone | Fluorochrome | Catalog | Vendor | Concentration | Purpose |  |
| --- | --- | --- | --- | --- | --- | --- | --- |
| CD3 | UCHT1 | APC | 555335 | BD | 1/30 | Lineage |  |
| CD56 | NCAM16.2 | APC | 555518 | BD | 1/900 |  |  |
| TCRγδ | B1 | APC | 331211 | Bio | 1/30 |  |  |
| CD4 | RPA-T4 | PE | 555347 | BD | 1/450 |  |  |
| CD8 | RPA-T8 | PE | 555367 | BD | 1/20 |  |  |
| CD14 | M5E2 | PE | 555398 | BD | 1/15 |  |  |
| CD19 | HIB19 | PE | 555413 | BD | 1/300 |  |  |
|  |  |  |  |  |  |  |  |
| CD4 | OKT4 | APC-eFluor780 | 47-0048 | eBio | 1/20 | Lineage equivalent |  |
| CD8 | HIT8a | FITC | 560960 | BD | 1/60 |  |  |
| CD20 | 2H7 | BV605 | 302333 | Bio | 1/60 |  |  |
| CD56 | REA-196 | PE-Vio770 | 130-098-132 | Milt | 1/30 |  |  |
| CD64 | 10.1 | BV510 | 305027 | BD | 1/30 |  |  |
| TCRαβ | IP26 | BV421 | 306721 | Bio | 1/20 |  |  |
| TCRγδ | B1.1 | PerCP-eFluor710 | 46-9959 | eBio | 1/30 |  |  |
|  |  |  |  |  |  |  |  |
| Dead cells |  | L/D Blue | L-23105 | LT | 1/300 | Live/Dead discrimination |  |

BD = BD Biosciences, Bio = BioLegend, eBio = eBioscience, Milt = Miltenyi, LT = Life Technologies

Antibody panel used to evaluate the expression of lineage markers using a classical seven fluorochrome approach.

| Target | Clone | Fluorochrome | Catalog | Vendor | Concentration | Purpose |
| --- | --- | --- | --- | --- | --- | --- |
| CD3 | UCHT1 | BUV737 | 564308 | BD | 1/30 | Lineage |
| CD4 | OKT4 | APC-eFluor780 | 47-0048 | eBio | 1/20 |  |
| CD8 | HIT8a | FITC | 560960 | BD | 1/60 |  |
| CD14 | M5E2 | PerCP-Cy5.5 | 340585 | BD | 1/15 |  |
| CD19 | HIB19 | PE | 555413 | BD | 1/300 |  |
| CD56 | NCAM16.2 | BV421 | 62751 | BD | 1/900 |  |
| TCRγδ | B1 | APC | 331211 | Bio | 1/30 |  |
|  |  |  |  |  |  |  |
| Dead cells |  | L/D Blue | L-23105 | LT | 1/300 | Live/Dead discrimination |

BD = BD Biosciences, Bio = BioLegend, eBio = eBioscience, Milt = Miltenyi, LT = Life Technologies

Antibody panel used to stain frozen PBMC from a patient with multiple myeloma.

| Target | Clone | Fluorochrome | Catalog | Vendor | Concentration | Purpose |  |
| --- | --- | --- | --- | --- | --- | --- | --- |
| CD3 | UCHT1 | BV421 | 562426 | BD | 1/20 | Lineage |  |
| CD56 | NCAM16.2 | BV421 | 562751 | BD | 1/900 |  |  |
| TCRγδ | B1 | BV421 | 331217 | Bio | 1/30 |  |  |
| CD4 | RPA-T4 | PE | 555347 | BD | 1/450 |  |  |
| CD8 | RPA-T8 | PE | 555367 | BD | 1/20 |  |  |
| CD14 | M5E2 | PE | 555398 | BD | 1/15 |  |  |
| CD19 | HIB19 | PE | 555413 | BD | 1/300 |  |  |
|  |  |  |  |  |  |  |  |
| CCR7 | G043H7 | AF647 | 353217 | Bio | 1/30 | Differentiation |  |
| CD45RA | HI100 | APC-H7 | 560674 | BD | 1/60 |  |  |
|  |  |  |  |  |  |  |  |
| CCR4 | 1G1 | PE-Cy7 | 561034 | BD | 1/60 | Th subsets |  |
| CCR6 | G034-E3 | BV605 | 353419 | Bio | 1/30 |  |  |
| CXCR3 | 1C6/CXCR3 | AF488 | 561730 | BD | 1/30 |  |  |
|  |  |  |  |  |  |  |  |
| CD57 | NK-1 | PE-CF594 | 562488 | BD | 1/900 | Activation/Exhaustion |  |
| HLA-DR | G46-6 | BV510 | 563083 | BD | 1/30 |  |  |
|  |  |  |  |  |  |  |  |
| CD16 | 3G8 | BUV395 | 563784 | BD | 1/30 | NK, Monocyte activation |  |
|  |  |  |  |  |  |  |  |
| Dead cells |  | L/D Blue | L-23105 | LT | 1/300 | Live/Dead discrimination |  |

BD = BD Biosciences, Bio = BioLegend, eBio = eBioscience, Milt = Miltenyi, LT = Life Technologies
